# Poly(l-lactic acid) Scaffold Releasing an α_4_β_1_ Integrin Agonist Promotes Nonfibrotic Skin Wound Healing in Diabetic Mice

**DOI:** 10.1021/acsabm.2c00890

**Published:** 2022-12-21

**Authors:** Vito Antonio Baldassarro, Valentina Giraldi, Alessandro Giuliani, Marzia Moretti, Giorgia Pagnotta, Alessandra Flagelli, Paolo Clavenzani, Luca Lorenzini, Luciana Giardino, Maria Letizia Focarete, Daria Giacomini, Laura Calzà

**Affiliations:** † Department of Veterinary Medical Science, University of Bologna, 50 Via Tolara di Sopra, 40064 Ozzano Emilia, Bologna, Italy; ‡ Interdepartmental Center for Industrial Research in Health Sciences and Technologies, University of Bologna, 41/E Via Tolara di Sopra, 40064 Ozzano Emilia, Bologna, Italy; § Department of Chemistry “Giacomo Ciamician” and INSTM UdR of Bologna, University of Bologna, 2 via Selmi, 40126 Bologna, Italy; ∥ IRET Foundation, 41/E Via Tolara di Sopra, 40064 Ozzano Emilia, Bologna, Italy; ⊥ Department of Pharmacy and BioTechnology, University of Bologna, 15 Via San Donato, 40127 Bologna, Italy

**Keywords:** skin wound healing, diabetic ulcer, mice, PLLA electrospinning, α4β1 integrin

## Abstract

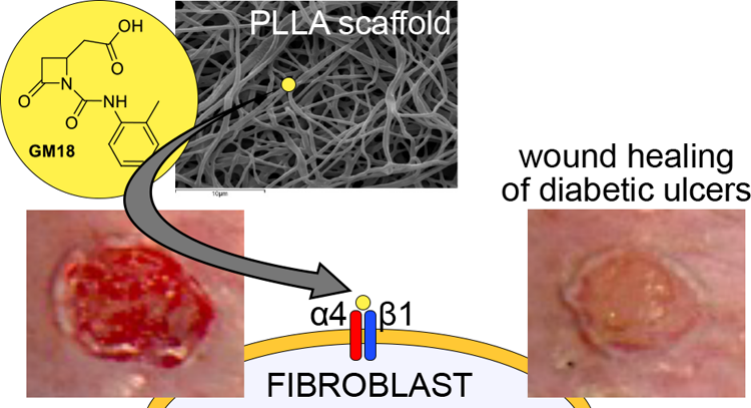

Skin wound healing is a highly complex process that continues to represent a major medical problem, due to chronic nonhealing wounds in several classes of patients and to possible fibrotic complications, which compromise the function of the dermis. Integrins are transmembrane receptors that play key roles in this process and that offer a recognized druggable target. Our group recently synthesized GM18, a specific agonist for α4β1, an integrin that plays a role in skin immunity and in the migration of neutrophils, also regulating the differentiated state of fibroblasts. GM18 can be combined with poly(l-lactic acid) (PLLA) nanofibers to provide a controlled release of this agonist, resulting in a medication particularly suitable for skin wounds. In this study, we first optimized a GM18-PLLA nanofiber combination with a 7-day sustained release for use as skin wound medication. When tested in an experimental pressure ulcer in diabetic mice, a model for chronic nonhealing wounds, both soluble and GM18-PLLA formulations accelerated wound healing, as well as regulated extracellular matrix synthesis toward a nonfibrotic molecular signature. In vitro experiments using the adhesion test showed fibroblasts to be a principal GM18 cellular target, which we then used as an in vitro model to explore possible mechanisms of GM18 action. Our results suggest that the observed antifibrotic behavior of GM18 may exert a dual action on fibroblasts at the α4β1 binding site and that GM18 may prevent profibrotic EDA-fibronectin-α4β1 binding and activate outside-in signaling of the ERK1/2 pathways, a critical component of the wound healing process.

## Introduction

Skin wounds still represent a major medical problem in conditions characterized by chronic nonhealing wounds,^1^ in surgical wound complications, and in elderly bedridden individuals.^2^ Chronic disease can also lengthen healing time and favor the onset of complications. Approximately 25% of people with type 2 diabetes experience foot ulcer, and their risk of amputation is 10–20 times higher than in people without the condition.^3^ Skin repair, although complete, may be characterized by a pathological evolution, such as fibrosis due to the accumulation of extracellular matrix (ECM), or an insufficient remodeling phase, leading to compromised function and an altered architecture of the dermis.^4^ Transforming growth factor β (TGFβ) signaling is the major activator of fibroblasts, myofibroblasts, and collagen synthesis in these conditions.^5^ Chronic hypertrophic scarring may also lead to the formation of keloids—pathological scars that grow over time and extend beyond the initial site of injury, causing pain, itching, and discomfort.^6^ Treatment aimed at promoting skin wound repair, therefore, should ideally not only reduce healing time but also promote proper tissue restoration. One recognized target for this purpose is the interaction between the cells involved in the healing process, such as fibroblasts and endothelial cells, and proper ECM production.^7^

Integrins are key molecules in these processes,^8^ acting not only as adhesion receptors for cell–cell and cell–matrix interactions but also as outside-in signaling, regulating migration, survival, and growth. They are grouped into four subfamilies of 24 αβ heterodimeric members composed of noncovalently associated α and β subunits, based on their ligand specificity or phylogenetic comparison of the α unit. α_4_β_1_, together with α_4_β_7_ and α_9_β_1_, is a component of the subfamily of integrins that recognize ECM ligands in an arginine–glycine–aspartic acid (RGD)-independent manner, meaning that they bind ECM proteins such as fibronectin (FN), via adhesive sequences EILDV and REDV,^9^ rather than through the RGD fragment (arginine, glycine, and aspartic acid).

Integrins are recognized pharmacological targets in many pathological conditions, and selective ligands for the different heterodimers are under active investigation.^10^ We recently developed monocyclic β-lactam derivatives with an amine, a carboxylate side chain, and the β-lactam ring as a site of conformational restriction.^11^ In the relative library, potent agonists which may induce cell adhesion and promote cell signaling mediated by integrins α_v_β_3_, α_v_β_5_, α_5_β_1_, or α_4_β_1_ were successfully obtained. Of these compounds, GM18 is a specific agonist for the α_4_β_1_ integrin, a dimer composed of CD49d (α4) and CD29 (β1), whose primary ligands include VCAM-1 and fibronectin. GM18 promotes human bone marrow mesenchymal stem cell (MSC) adhesion and viability in vitro, as well as differentiation toward osteoblastic lineage increases in co-cultures of human primary mesenchymal stem cells (hMSCs) and human primary osteoclasts (OCs),^12^ promoting cell adhesion by driving changes in focal adhesion protein distribution (β_1_ integrin and vinculin) and activation (pFAK).^13^ GM18 has also been successfully combined with poly(l-lactic acid) (PLLA) nanofibers to provide a controlled release of this agonist, resulting in a medication particularly suited to skin wounds.^13^

The integrin α_4_β_1_ plays a role in skin pathophysiology, where it forms part of the skin immune response, promotes the migration of neutrophils,^14^ and regulates the differentiated state of fibroblasts based on microenvironmental cues.^15^ It is also expressed in endothelial progenitor cells^16^ and the vascular endothelium where it binds to VCAM-1, mediating leukocyte trafficking to sites of inflammation.^17^

Based on these characteristics of α_4_β_1_, we decided to further explore the possible use of GM18 in skin wound healing. We performed three sets of experiments: in vitro experiments to fully characterize the release of GM18 from the PLLA scaffold; in vivo experiments in the pressure ulcer (PrU) model in diabetic mice to establish the effectiveness of PLLA-GM18 in the wound healing timeline and related molecular profile; and cell culture experiments to test GM18 activity in specific cells involved in wound healing—human fibroblasts, keratinocytes, and endothelial cells.

## Results

**Figure 1:**
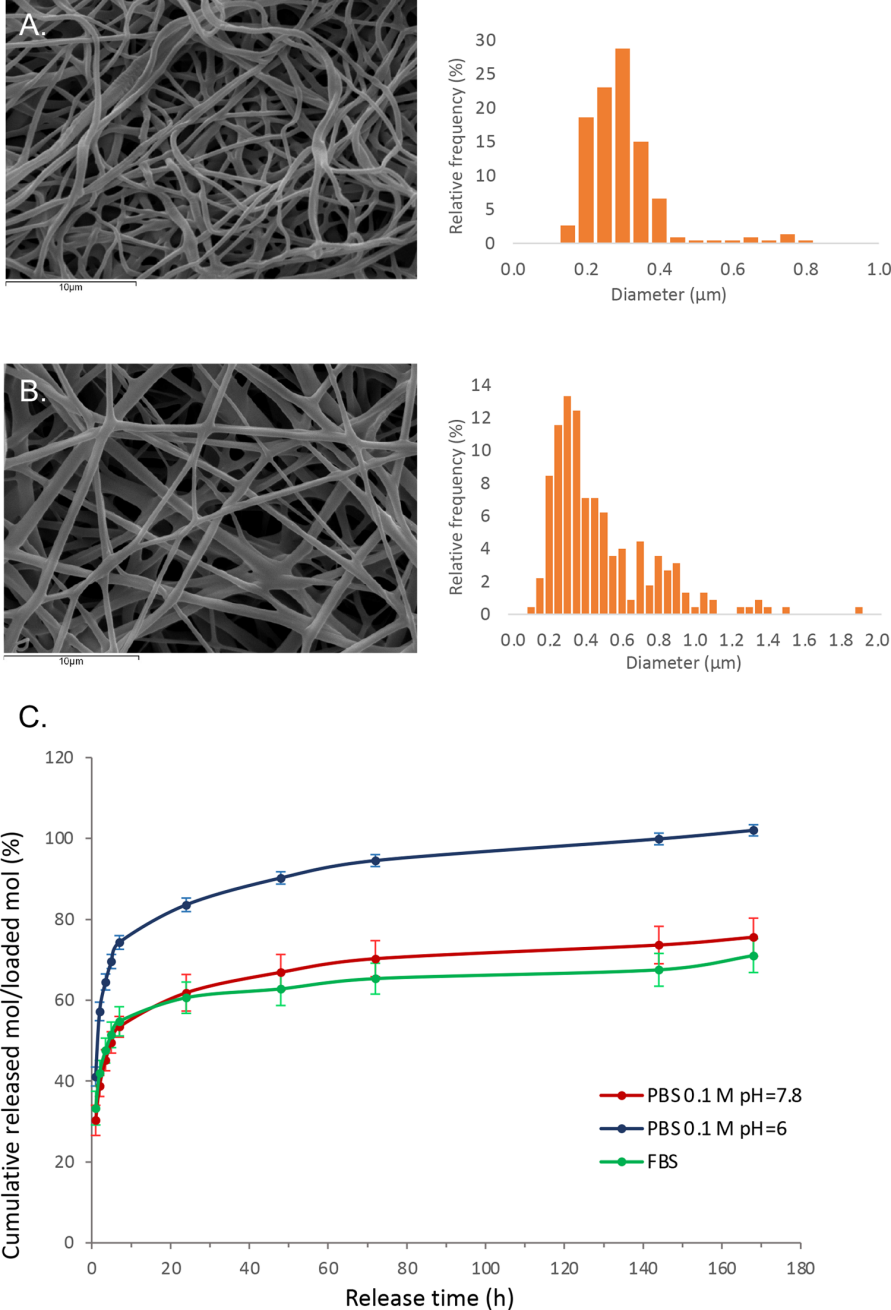
Characterization of the GM18-loaded scaffolds. Scanning electron microscopy images (4000×) (left) and fiber diameter distribution (right) of (A) PLLA5GM18 and (B) PLLA15GM18. (C) Release of GM18 from the PLLA15GM18 scaffold in 0.1 M phosphate-buffered saline (PBS) at pH 7.8 (red), 0.1 M PBS at pH 6 (blue), and fetal bovine serum (FBS) (green). The cumulative release is reported as % of released mol compared to the actual loaded mol over release time (hours). The bars represent the mean ± standard deviation (SD of the mean values) in triplicate.

In this study, we aimed to expand our previous research on a PLLA electrospun scaffold combined with the selective integrin ligand GM18,^13^ by investigating the application of the PLLA-GM18 scaffold in wound healing. The previously optimized methodology, starting from a blend of the polymer and GM18, is easy and reproducible, and results in randomly oriented, bead-free nanofibers, as shown in the scanning electron microscopy images for the two scaffolds containing 5 and 15 wt % of GM18 (Figure 1). The average fiber diameter was 0.32 ± 0.10 μm for PLLA5GM18, and 0.51 ± 0.29 μm for PLLA15GM18, with a broader diameter distribution and less homogeneous fibers in the latter.

Differential scanning calorimetry (DSC) analysis reveals that GM18 had a plasticizing effect on PLLA, decreasing the glass-transition temperature from 58 °C (plain PLLA) to 48 °C (PLLA15GM18), and reducing its degree of polymer crystallization in a GM18 concentration-dependent manner. The calorimetric results are consistent with previously reported data for PLLA-GM18 containing 10 wt % of GM18,^13^ and explain the higher average fiber diameter found for the more plasticized scaffold.

The actual amount of GM18 inside the scaffolds was 4.35 ± 0.6 and 12.2 ± 0.1% for the PLLA5GM18 and PLLA15GM18 scaffolds, respectively, results consistent with those previously reported for the 10 wt % PLLA-GM18 mat, where the actual amount of loaded GM18 was determined to be 7.48 wt %.^13^

### GM18 Release from the PLLA Scaffold

The GM18 release studies were conducted on a dry PLLA15GM18 mat, the type of scaffold chosen for the in vitro and in vivo tests. The PLLA15GM18 mat is easily wettable, probably due to the presence of GM18 which increases its hydrophilicity (as opposed to the plain PLLA mat, which is hydrophobic and requires a prewetting treatment to enhance cell interaction with the scaffold^18^), therefore the mat was used in its dry form, without any prewetting treatment. The release profiles of GM18 were evaluated in three different mediums as models of different physiological conditions. It is known that pH changes occur in the presence of a wound,^19^ and by choosing PBS at different pH levels, i.e., slightly basic (pH 7.8) and acidic (pH 6.0), we wanted to evaluate how release is affected by pH. FBS, on the other hand, which provides an environment rich in proteins, enzymes, electrolytes, and nutrients, was evaluated as a model of wound exudate. The cumulative released mol % of GM18 with respect to the nominal loaded amount was plotted against the release time to obtain GM18 release profiles, shown in Figure 1C. The three release profiles share a similar pattern, with an initial burst release followed by a slow-release phase after the fifth medium change (7 h) (Figure 1C). The faster initial release kinetics is related to the compound loaded in the external part of the fibers, while the innermost GM18 meets diffusional limits and is therefore related to the slow sustained release observed over time. During the first five medium changes, 55 and 53% of loaded GM18 were released into FBS and PBS (both at pH 7.8), respectively, reaching 74% in the case of PBS at pH 6. After 7 days and 10 medium changes, the entire amount of loaded GM18 was released into the acidic environment of PBS at pH 6, a considerably higher amount compared to those released into PBS at pH 7.8 and FBS (76 and 71%, respectively). This can be explained by a higher degradation rate of PLLA nanofibers in acidic conditions, with the consequent release of encapsulated GM18.

### Effectiveness of GM18 and PLLA-GM18 in Wound Healing Timeline and Related Molecular Profile

**Figure 2:**
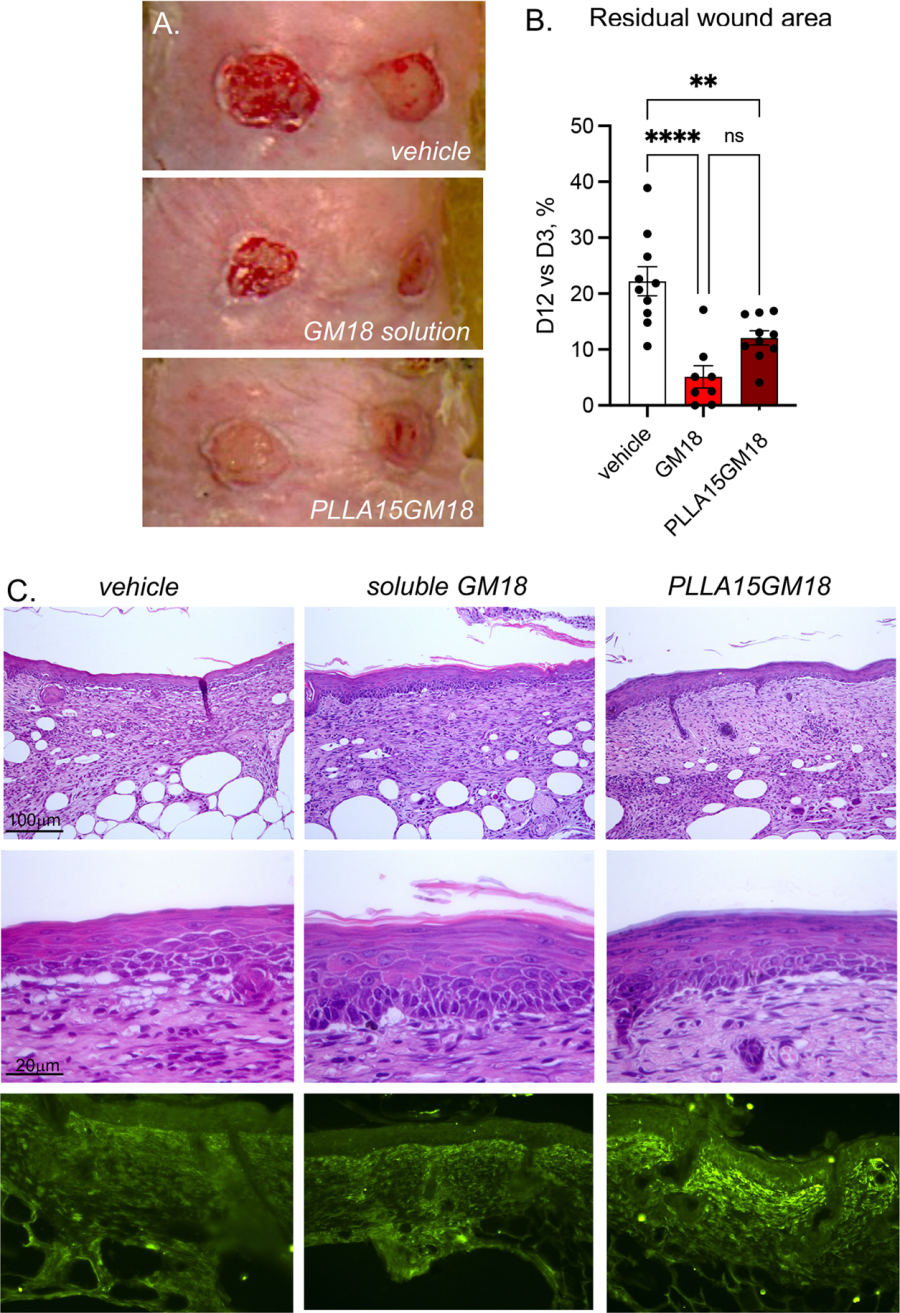
Effect of soluble GM18 and GM18-loaded scaffold treatments on pressure ulcer. (A) Representative photos of the pressure ulcers on day 14 following wound medication in db/db mice medicated with vehicle, GM18 solution, and PLLA-GM18. (B) Ulcer areas in the experimental groups over the observational days. *N* = 12 ulcers/group were included in this experiment. Statistical analysis: one-way analysis of variance (ANOVA), **p < 0.01, ****p < 0.0001. (C) Hematoxylin and eosin (H&E) histological staining of the already reepithelized area around the wound at low (upper micrographs)- and high-magnification (intermediate micrographs). Vimentin-immunostaining is also presented (bottom micrographs).

We then studied the effects of topical GM18 application on wound healing and related molecular machinery, applied in solution or loaded in PLLA. PLLA loaded with 15% GM18 (PLLA15GM18) was chosen, based on the results of the GM18 release tests and from previous in vitro studies on mesenchymal stem cells.^20^ This experiment was conducted in the pressure ulcer model in diabetic mice, as already established and characterized in our lab.^21,22^ Photos of representative ulcers from the three experimental groups at 14 days from the first medication are shown in Figure 2A. The repaired area was measured as % value on day 14 compared to day 3 (when surgical curettage and the first medication were performed) and expressed as “residual wound area”. The results in the graphs show that GM18 promotes wound reepithelization, both when used in solution and as PLLA15GM18 (Figure 2B). The epithelial layer is also thicker and better-structured in GM18 and PLLA15GM18 mice, where the different cell types of the multilayer epithelium are already evident (Figure 2C). Moreover, vimentin-IR strongly increases in GM18 and PLLA, suggesting a greater fibroblast recruitment by experimental medications.

To explore the molecular signature of the tissue repair, we analyzed the expression level of 84 genes encoding for proteins of the extracellular matrix, in treated mice compared to vehicle.

**Figure 3:**
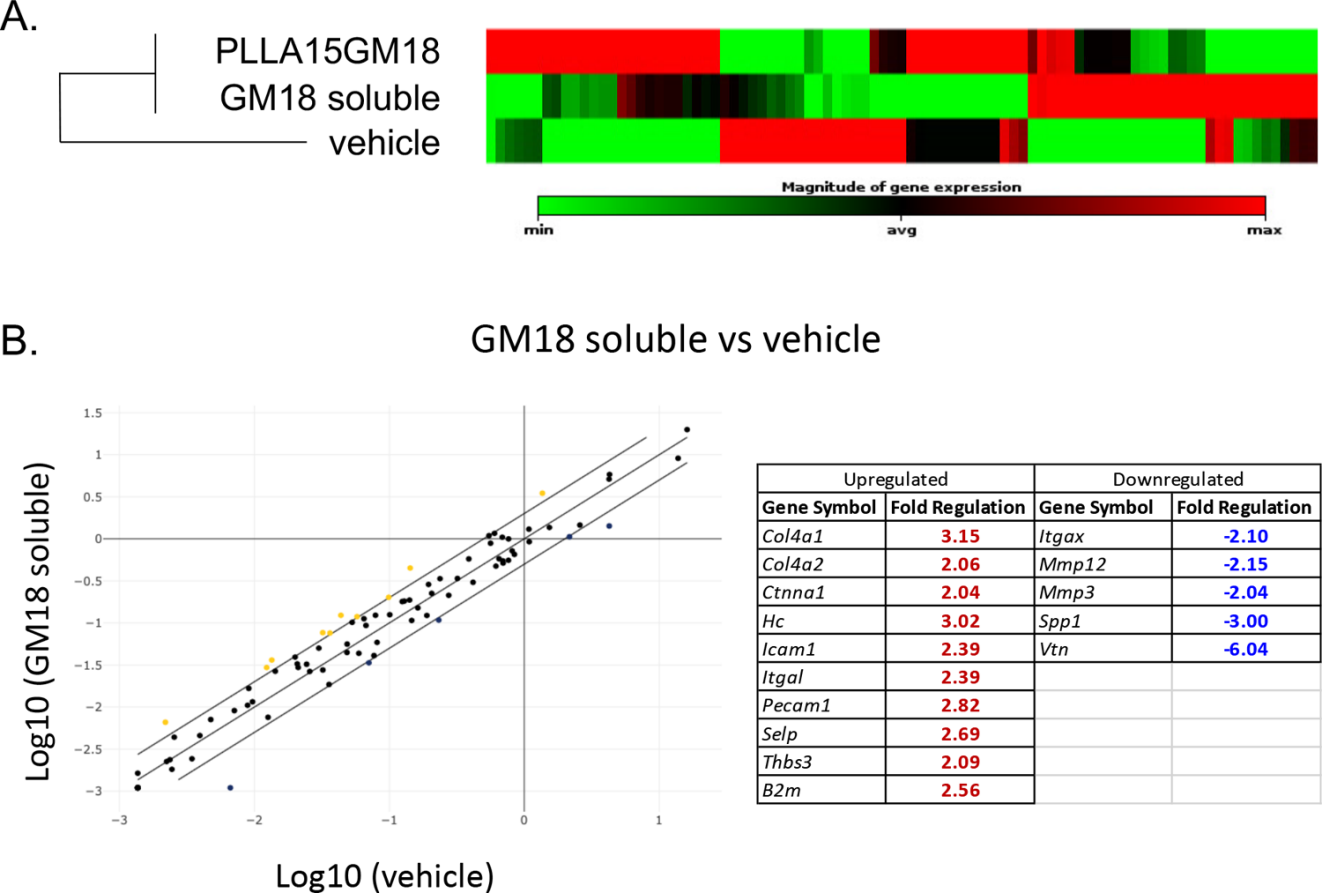
Effect of GM18 treatments on extracellular matrix-related genes in db/db PrU ulcers. (A) Clustergram showing the clusterization of the three experimental groups. The magnitude of expression is indicated with a color code between the maximum (red) and minimum (green) of expression within the same gene across the groups. (B) Graph showing the scatter plot of the relative gene expression regulation of tissues treated with GM18 as a soluble molecule versus tissues treated with GM18-loaded scaffolds, with a cutoff value of fold of changes >2. The regulated genes, with the relative fold of change values, are shown in the table, indicating upregulated (red) and downregulated (blue) genes in GM18-treated tissues compared to vehicle.

The genes included in the analysis encode for cell adhesion molecules (transmembrane receptors, cell–cell adhesion, cell–extracellular matrix adhesion), extracellular matrix molecules (basement membrane constituents, collagens, and ECM structural constituents, extracellular matrix proteases, and protease inhibitors; see Table S2 for the full gene list). The relative quantification of mRNA expression was calculated using the comparative cycle threshold (Ct) method and is shown as a heat map of the twofold hierarchical clustering, showing correlated gene expression across each group with red being the maximum and green the minimum difference of expression from the median of each gene analyzed (Figure 3A).

According to the data analysis software, the geometric mean of the *Actb*, *Hprt1*, and *Gapdh* gene expression was used as housekeeping for the normalization procedure. This analysis indicated that the expression profiles of the ECM encoding genes in GM18- and PLLA15GM18-treated mice were similar and different from vehicle-treated mice (Figure 3A; the complete clustergram is shown in Figure S4).

The analytical results of GM18 vs vehicle are presented in Figure 3B as a scatter plot analysis, and the relative fold change for each gene significantly regulated (>2-fold of increase) is shown in the table. Upregulated genes include *Col4a1* and *Col4a2*, encoding for collagen type IV α 1 and 2 chains; *Ctnna1*, encoding for catenin α 1; *Hc*, encoding for hemolytic complement; *Icam1*, encoding for intercellular adhesion molecule 1; *Itgal*, encoding for integrin subunit α L; *Pecam1*, encoding for platelet endothelial cell adhesion molecule 1 or CD31; *Selp*, encoding for Selectin P; *Thbs3*, encoding for thrombospondin 3, and *B2n*, encoding for β 2 microglobulin. Downregulated genes include *Itgax*, encoding for integrin subunit α X; *Mmp3* and *Mmp12*, encoding for metalloproteinase-3 and 12; *Spp1*, encoding for secreted phosphoprotein 1, and *Vtn*, encoding for vitronectin.

To better analyze if the delivery solution affects ECM molecular regulation, we also compared soluble GM18 to PLLA15GM18, and the results are shown in Figure S5 (Supporting Information Section S4) as a scatter plot (A, B), where the table summarizes the upregulated (red) and downregulated genes (blue). While the same genes were either regulated or nonregulated according to the GM18 administration strategy, the vitronectin encoding gene was down- or upregulated by soluble GM18 or PLLA15GM18, respectively (Figure S6).

### GM18 on the Adhesion Properties on Skin Cells

**Figure 4:**
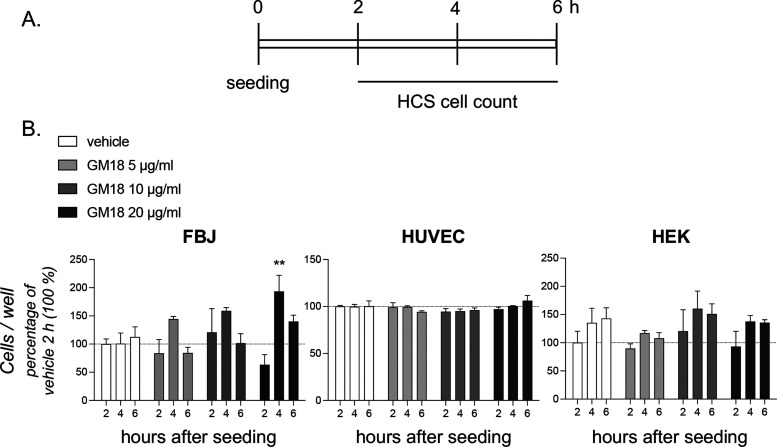
Effect of GM18 coating on fibroblasts, endothelial cells, and keratinocyte cell adhesion. (A) Experimental procedure. Cells were seeded on 96-well plates coated with GM18 at three different concentrations (5, 10, and 20 μg/mL). At different indicated time points, according to the standard adhesion timing of the different cell lines, the cultures were fixed, nuclei were stained with Hoechst nuclear staining, and the total number of cells per well was measured by cell-based high-content screening (HCS). (B) Graphs showing the total cell number per well of the different cell lines at the indicated time points. Data are represented as percentage of the control group (vehicle, 2 h = 100%). Statistical analysis. Columns represent the mean ± standard error of the mean (SEM). One-way ANOVA followed by Dunnett’s post-test. Asterisks represent a statistically significant difference (**p < 0.01).

To dissect the main cell target for GM18 in the skin, we tested the GM18 binding capacity of the three main cell types involved in wound healing—endothelial cells (human umbilical vein endothelial cell (HUVEC)), fibroblasts (FBJ), and keratinocytes (HEK). A conventional passive in vitro cell adhesion test was performed to explore phase I of the adhesion stages (Khalili and Ahmad, 2015)^100^ using a GM18-coated substrate, and the cell body attachment to GM18 measured at short time points from seeding (2, 4 and 6 h, Figure 4A for the test schedule). Only fibroblasts were sensitive to the presence of the molecule on the adhesion substrate in these experimental conditions, showing an increase in cell adhesion, with a statistical significance at 4 h after seeding (Figure 4B). We therefore selected these cells for a more thorough analysis.

**Figure 5:**
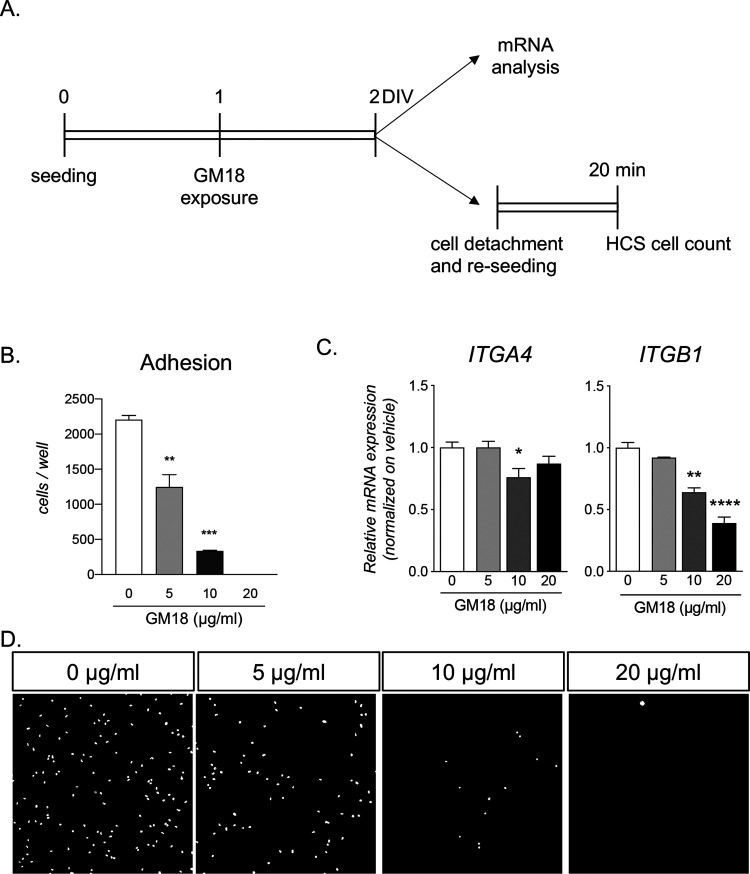
Effect of GM18 exposure on gene expression and adhesion capacity of fibroblasts. (A) Experimental procedure. One day after seeding, the cells were exposed to three different concentrations of GM18 (5, 10, and 20 μg/mL) and lysed for gene expression analysis after 24 h. In another set of experiments, following the 24 h exposure, the cells were detached and seeded again. After 20 min, the total number of cells in each well was analyzed using the cell-based high-content screening (HCS) technology based on nuclear staining. (B) Graphs showing the relative expression of ITGA4 and ITGB1 genes in vehicle-treated groups (white column, 0 μg/mL). (C) Graph showing the total cell count per well, 20 min after seeding. Statistical analysis. Columns represent the mean ± SEM. One-way ANOVA followed by Dunnett’s post-test. Asterisks indicate the statistically significant differences (*p < 0.05; **p < 0.01; ***p < 0.001; ****p < 0.0001).

We then tested if the binding of GM18 to the target site modified cell adhesion. For this test, cells were exposed to GM18 for 24 h, detached, and reseeded. To analyze early adhesion, cultures were blocked 20 min after seeding and stained with Hoechst to count the total number of cells in each well (Figure 5A). FBJ fibroblasts exposed to GM18 showed a dramatic decrease in cell adhesion (one-way ANOVA, *F*(2.5) = 51.24, *p* = 0.0005), resulting in a small number of cells attached at 5 μg/mL (Dunnett’s post-test, *p* = 0.0040) and 10 μg/mL (*p* = 0.0003), while no cells were detected at 20 μg/mL (Figure 5B).

To explore the inside-down effects of the 24 h exposure to the α_4_β_1_ ligand GM18 on FBJ cells, we analyzed the mRNA expression of the two target integrins (Figure 5C,D). *ITGA4* was significantly decreased (one-way ANOVA, *F*(3.8) = 6.206; *p* = 0.0175) at a dose of 10 μg/mL (Dunnett’s post-test, *p* = 0.0175) as was *ITGB1* gene expression (one-way ANOVA, *F*(3,8) = 54.15; *p* < 0.0001) at the two higher doses (Dunnett’s post-test, 10 μg/mL, *p* = 0.0012; 20 μg/mL, *p* < 0.0001), showing a sensitivity of the fibroblast cell line FBJ to GM18 exposure in terms of gene expression regulation.

**Figure 6:**
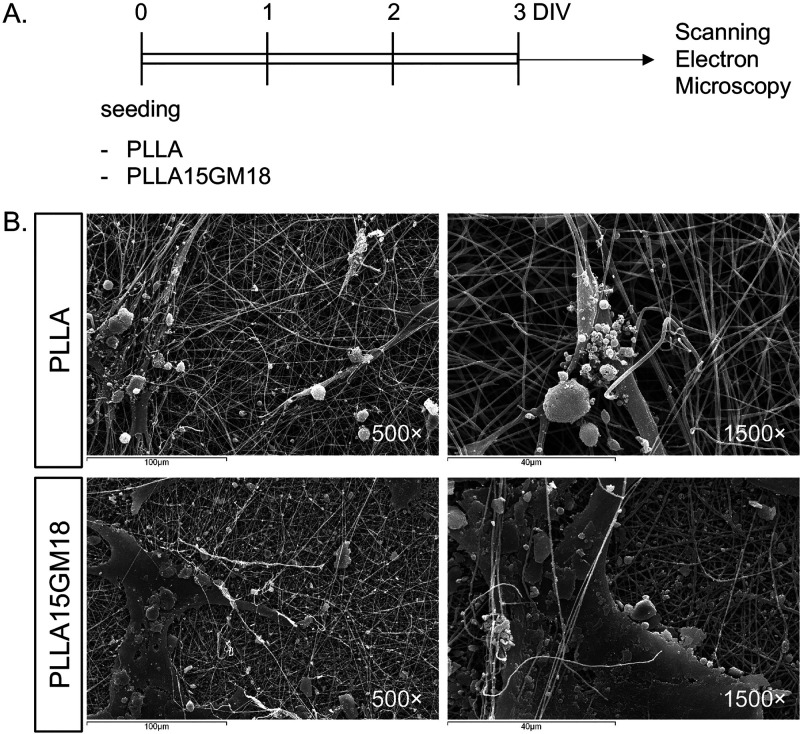
Effect of GM18-loaded scaffolds on fibroblast cell morphology. (A) Experimental procedure. FBJ cells were seeded on PLLA or PLLA15GM18 scaffolds and, after 3 days in vitro (DIV), were fixed and processed for the scanning electron microscope acquisition. (B) Representative pictures of Scanning electron microscope images of FBJ cells seeded on PLLA or PLLA15GM18 scaffolds. Scale bars are included in each image.

As a preliminary investigation of the direct effect of the scaffold on the cell response, we seeded the FBJ cells on PLLA and PLLA15GM18 scaffolds to visualize the cell morphology through scanning electron microscopy (Figure 6A). The images show how the cells adhere and integrate with the PLLA fibers, a dynamic possibly boosted by the presence of the GM18 molecule enhancing a wider expansion of the cell body (Figure 6B).

## Discussion

Regulation: The α_4_β_1_ integrin has a well-established role in leukocyte biology and is already a recognized target for pharmacological intervention; in fact, natalizumab, a pan-α_4_ antagonist and α_4_β_1_ ligand binding inhibitor, is an approved therapy in Crohn’s disease and multiple sclerosis, for its ability to reduce trans-barrier leukocyte transmigration.^10^ The biology of integrins, however, is extremely complex, and their conformation can change according to the microenvironmental conditions, affecting their binding capacity^23^ and their pharmacological modulation by different classes of ligands as a result.

GM18 is a β-lactam derivative designed and synthesized to target the integrin receptor site of the tripeptide arginine–glycine–aspartic acid (RGD),^11^ and has been characterized for its biological effects on cell culture systems. When tested in circulating cells, such as Jurkat E6.1 cells (leukemic T-cell lymphoblast), GM18 increases cell adhesion by acting as an agonist via ERK1/2 phosphorylation.^11^ Researchers have also embedded GM18 in a PLLA scaffold, resulting in a progressive and sustained release that promoted the adhesion of bone marrow-derived mesenchymal stromal cells.^13^

In this study, we explored the use of GM18 embedded in a polymeric scaffold as a medication for skin wound healing in diabetic mice. Skin wound healing is a complex biological process tightly regulated at cellular and molecular level^24^ and consisting of four phases. The first is coagulation, beginning at the onset of injury, and aimed to stop bleeding, and the interaction of platelets with the extracellular matrix promotes clot formation. The subsequent phase is inflammation, focused on destroying bacteria and removing debris, to prepare proliferation. The proliferation phase includes filling the wound by fibroblast migration and extracellular matrix production, contraction of the wound margins; and finally reepithelization, to cover the wound. The last phase is remodeling, or maturation phase, during which the new tissue slowly gains strength and flexibility. An inappropriate remodeling leads to a fibrotic scar. During these phases, interrelated and overlapping mechanisms of cell migration and proliferation, synthesis of extracellular matrix, growth factors and cytokines coordinate the healing process,^25^ and integrins are involved in almost all of these steps.

For this in vivo study, we used a model of skin wound currently used by researchers to mimic chronic, nonhealing wounds, consisting of a pressure ulcer in db/db mice, a widely used mouse model of type 2 diabetes mellitus.^26^ These mice develop progressive sensory loss, electrophysiological impairments, and skin innervation loss:^27^ the skin repair process is also much slower in db/db than in control mice,^22^ making this mouse model highly appropriate for testing pro-healing compounds.

GM18 accelerates wound healing and increases epithelial thickness, both in solution and 15% PLLA scaffold form. None of the ulcers in vehicle-treated mice were closed at observation time (14 days after the start of treatment) and the mean area of the open wound was around 22%, while this area was less than 5% in mice treated with soluble GM18 and 11% in mice treated with PLLA15GM18.

Since various cell types are involved in this process, we attempted to establish the preferential GM18 target cell. We first screened three types of cells (human endothelial cells, fibroblasts, and keratinocytes) to identify the type most responsive to GM18, using the cell adhesion test on GM18-coated wells, a cell assay that has proven highly sensitive to the biological effects of GM18. The presence of GM18 on the substrate increased dermal fibroblasts, but not endothelial and keratinocyte adhesion, therefore we focused on the former for further analysis.

Fibroblasts are the main cell type responsible for ECM deposition during wound healing: fibroblast dysfunction has also been identified as a factor in fibrotic scar and keloid formation.^28^ We then studied the expression level of 83 genes encoding for cell adhesion molecules and extracellular matrix molecules, most of them associated with fibroblasts (basement membrane constituents, collagens & ECM structural constituents, ECM proteases, and ECM protease inhibitors). Overall, 10 genes are upregulated and 5 are downregulated by GM18 treatment. Upregulated genes include several pro-healing genes, such as *Pecam1*, a marker for the capillary network; *Col4a1* and *Col4a2* which make up the basal membrane and guarantee its integrity;^29^*Icam1* and *Selp*, whose deletion delays wound healing,^30^ and *Ctnna1* (αE-catenin), which plays a role in epithelial tissue, both at adherens junctions and in signaling pathways.^31^ Notably, several downregulated genes in GM18-treated mice are associated with skin fibrosis: *Itgax* encodes for the integrin α X (CD11c), while ITGAX/CD11c are markers for inflammation-driven fibrosis.^32^*Spp1* (osteopontin) acts upstream of TGFβ to promote fibrosis,^33^ while *Mmp12* and *Mmp3* are listed among profibrotic enzymes in the dermis,^34^ suggesting that GM18 promotes an antifibrotic phenotype of fibroblasts. This result is remarkable since uncontrolled tissue fibrosis leads to pathological and painful scars, including keloids, and much less attention is dedicated to appropriate scar formation, compared to the more general healing effects, as established by reepithelization. Biomaterial having different adhesive or mechanomodulatory properties also conjugated with bioactive molecules has been proposed to prevent fibrotic scar formation.^35^ Here we propose a highly selective biological target, i.e., α_4_β_1_ mediated effects, having biomaterial as a key component allowing appropriate release kinetic.

This antifibrotic behavior of GM18 may be associated with a dual action at the binding site. By occupying the α_4_β_1_ binding site on fibroblasts, GM18 interferes with the fibroblast-ECM interaction: in particular, α_4_β_1_ appears to bind a specific isoform of fibronectins (FNs), glycoproteins of the ECM, namely, the cellular form (cFN), which contains both EDA and EDB domains (EDA-FN). This FN isoform is produced by fibroblasts, epithelial or other resident cells, and deposited as fibrils in the ECM.^36^ EDA-FN is essential for maintaining proper skin wound healing, as also demonstrated by knock-out mice which display abnormal wound healing compared to Wt.^37^ The EDA insert of the cFN produced at the injury site triggers pathways that induce inflammation and increase ECM deposition and the activation of fibroblasts. This protein is also highly concentrated in keloid scars where they sustain inflammation and the production of collagen.^38^ The interaction between EDA-FN and the α_4_β_1_ integrin receptor promotes a profibrotic contractile phenotype in dermal fibroblasts, characterized by an increase in actin stress fibers, myosin light chain phosphorylation, and FN production.^39^ α_4_β_1_ is also the main regulator of EDA-FN-dependent synthesis of cytokines by fibroblasts.^39,40^

One possible theory is that the GM18 progressively released by the PLLA scaffold in vivo occupies the α_4_β_1_ binding site, thus preventing EDA-FN-α_4_β_1_ binding, and mitigating the profibrotic effect of this interaction. In fact, integrin α_4_β_1_ controls fibroblast proliferation and TFGβ1 processing,^41^ supporting a mechanism that suppresses α-SMA expression, a key molecular event in fibrosis dependent on microenvironmental cues,^15^ regulating the phenotype in dermal fibroblasts.^39^

Our in vitro results support this hypothesis, indeed in vitro treatment of fibroblasts with GM18 leads to a loss of the adhesion properties of these cells, thus indicating a functional GM18 occupancy of the α_4_β_1_ binding site. A similar approach has been used with a polypeptide with a predicted high binding affinity to α_4_β_1_ (AF38P), which has been shown to bind specifically to myofibroblast fibronectin-rich ECM and EDA-FN. AF38P has demonstrated potent myofibroblast inhibitory activity, attenuating the expression of pro-matrix MMP9 and inhibiting collagen synthesis and deposition, thus blocking profibrotic cell activity.^42^

GM18 may also act as an α_4_β_1_ agonist to activate outside-in signaling pathways via ERK1/2.^11^ This pathway plays an essential role in skin repair, and ERK activation promotes wound healing by accelerating the migration of keratinocytes,^43^ while inhibition of ERK phosphorylation has been shown to cause a dose-dependent delay of wound closure in the cornea.^44^ Notably, the up-regulation of ERK1/2 in human diabetic foot wounds is associated with a positive effect of negative pressure wound therapy (NPWT).^45^

It should be stressed that the in vitro and in vivo conditions presented in this study are quite different from each other. While cell adhesion appears to offer a standardized test, in reality, it reflects a complex phenomenon that changes over time, one in which integrin affinity and dynamics temporally evolve in the same experimental setup^46^ and the relative integrin affinity for the ligands depends on the equilibrium between different conformational states.^47,48^ Similarly, α_4_β_1_ plays different roles toward other cell types involved in the healing process, such as endothelial precursors, macrophages, and keratinocytes.

## Experimental Section

### GM18 Synthesis

Commercially available reagents and ACS-grade solvents were used without further purification. ^1^H NMR spectra were recorded with an INOVA 400 instrument using a 5 mm probe. All chemical shifts were quoted relative to deuterated solvent signals (δ in ppm and *J* in hertz). Merck 60 F254 thin-layer chromatography (TLC) plates were used to monitor the reactions. The GM18 compound was prepared starting from the commercially available 4-acetoxy azetidine-2-one, using a three-step synthesis (Scheme S1) previously optimized and reported^11^ (see Supporting Information Section S1 for detailed procedures and characterization). GM18 was obtained in high-purity grades (>95%), as assessed by high-performance liquid chromatography–mass spectrometry (HPLC-MS) analysis.

### Functionalized Scaffolds: Preparation and Characterization

Electrospinning was carried out with a homemade apparatus consisting of a high-tension voltage supply (Spellman SL 50 P10/CE 230), pump (KD Scientific 200 Series), and a glass syringe, connected to a needle (Hamilton NP3-G24, inner diameter of 0.54 mm) by a poly(tetrafluoroethylene) (PTFE) tube. The electrospinning apparatus was placed inside a glove box (Iteco Eng., Ravenna, Italy 100 × 7 × 100 cm^3^) fitted with a system to control temperature and humidity. The PLLA (Lacea H.100-E Mw 8.4 × 104 g/mol) was purchased from Mitsui Fine Chemicals (Düsseldorf, Germany), and commercially available reagents and ACS-grade solvents were used without further purification. PLLA-GM18 mats were obtained starting from a blend solution of GM18 and 13% w/v PLLA in a mixture of dichloromethane (DCM)/dimethylformamide (DMF) 65:35 v/v. To produce each mat, PLLA pellets (260 mg) were first dissolved in DCM (1.3 mL), then a solution of GM18 in DMF (0.7 mL) was added to form a homogeneous blend. Different loadings of GM18 (5 and 15 wt % with respect to the polymer) were achieved using 13.7 and 45.9 mg of GM18, respectively. The blend solutions were electrospun at room temperature, with a relative humidity of 50–60%, on a circular collector (10 cm diameter), with the following process parameters: applied voltage = 22 kV, flow rate = 1 mL/h and tip-collector distance = 15 cm. The scaffolds were dried on P_2_O_5_ under vacuum for three days to remove any solvent residue, sterilized by irradiation with γ rays, and stored at 4 °C. The obtained scaffolds were named PLLA5GM18 and PLLA15GM18 for PLLA containing 5 and 15 wt % GM18, respectively. Plain PLLA mats were prepared in the same way as a control without the addition of GM18.

### Scaffold Characterization

Differential scanning calorimeter (DSC) measurements were carried out using a TA Instruments Q2000 apparatus. A weighted sample (3–5 mg) was placed inside a Tzero aluminum pan and subjected to a heating scan at 20 °C/min from +20 to +200 °C, followed by quenching at +20 °C and then heating up to +200 °C at 20 °C/min, under nitrogen flow. Data were analyzed by TA Universal Analysis software.

Scanning electron microscopy images of the samples, sputter-coated with gold, were acquired using an INCAx-sight 7060 scanning electron microscopy apparatus with an accelerating voltage of 15 kV. The diameter of 225 fibers was then randomly measured on scanning electron microscopy images using ImageJ software. Statistics analysis (average diameter, standard deviation, and diameter distribution) was carried out using Excel.

### Determination of the Actual Amount of GM18 Loaded inside PLLA-GM18

The actual amount of GM18 inside the fibers was evaluated by HPLC analysis on PLLA5GM18 and PLLA15GM18 mats. An analytical HPLC apparatus was used with a Select Peptide CSH C18 3.5 μm 4.6 × 100 mm^2^ column at 30 °C, a 0.7 mL/min flow rate, and gradient elution from 70% H_2_O + trifluoroacetic acid (TFA) 0.08/30% acetonitrile (ACN) + TFA 0.08–30% H_2_O + TFA 0.08/70% ACN + TFA 0.08% in 12 min. A calibration curve was plotted starting from six GM18 standard solutions injected in triplicate into the analytical HPLC apparatus (GM18 retention time = 7.475 min) by correlating their concentrations with the average peak area determined at 254 nm. Three independent experiments were carried out for each type of PLLA-GM18 mat. Each mat sample was weighed on a precision balance and dissolved in 2 mL of DCM. Following solvent removal under reduced pressure, GM18 was quantitatively extracted by three successive washings with methanol (1 + 0.5 + 0.5 mL). The solution obtained (final volume = 2 mL) was then injected into the HPLC apparatus in triplicate. Concentrations were obtained by extrapolation from the calibration curve using the average peak areas. The extracted amount of GM18 was then compared with the theoretical amount inside the sample, calculated from the sample weight and the nominal amount of GM18. Results are expressed as the average between the three samples, with its standard deviation.

### Release Studies

The in vitro release profile of GM18 from PLLA15GM18 mat was evaluated via HPLC-MS analysis in phosphate-buffered saline (PBS) 0.1 M at pH = 7.8, PBS 0.1 M at pH = 6, and in fetal bovine serum (FBS). A UPLC-MS apparatus was used with an Agilent InfinitiLab Poroshell 120 EC-C18 3.0 × 150 mm^2^ 2.7 μm column, flow 0.4 mL/min at 40 °C; eluent phase from 70% H_2_O + TFA 0.1/30% MeCN + TFA 0.1–10% H_2_O + TFA 0.1/90% MeCN + TFA 0.1% in 12 min. Three mat samples for studies in PBS and two samples for FBS were weighed on a precision balance and used to carry out independent release experiments in their dry form, without any prewetting treatment. Each sample (1 × 1 cm^2^, weight ranging from 3 to 5 mg) was incubated at 37 °C in a thermostat with 0.5 mL of the release medium. At set time intervals (1, 2, 3.5, 5, 7, 24, 48, 72, 144, and 168 h), the supernatant was separated, conserved for HPLC-MS analysis, and replaced with 0.5 mL of fresh medium. For HPLC-MS analysis, the supernatants were diluted twice and injected directly into the HPLC-MS apparatus for PBS, while for FBS, 150 μL of the supernatants were first diluted with 0.6 mL of methanol, with subsequent precipitation of serum proteins, and then centrifuged at 55 000 rpm for 20 min. The clear liquid supernatant was then separated from the solid and analyzed by HPLC-MS. The calibration curve was obtained from seven GM18 standard solutions prepared in triplicate by correlating their concentrations with the corresponding average peak area determined at 254 nm (GM18 retention time = 5.57 min). Based on the dilution factors, GM18 concentration in the supernatants was determined by extrapolation from the calibration curve, giving the micrograms of GM18 released each time the medium was changed. Cumulative GM18 release was expressed as the percentage of the total released moles divided by the loaded moles, which in turn were calculated from the sample weight and the actual amount of GM18, determined as previously described.

### Animals

All animal protocols described herein were carried out according to the European Community Council Directives (2010/63/EU), complied with the ARRIVE guidelines and the NIH Guide for the Care and Use of Laboratory Animals, and were approved by the Ministry of Health (authorization no. 391/2017-PR). Ten- to eleven-week-old genetically diabetic C57BL/KsJ-m+/+Leprdb (db/db) male mice (Charles River Laboratories, Calco, Lecco) were included in the experiment. The animals were housed with food pellets and water ad libitum, and a dark–light cycle of 12 h. Blood glucose was measured prior to wound induction and prior to sacrifice in nonfasting animals, between 9 and 10 AM (Contour XT, Bayer, Basel, Switzerland). All mice included in the study showed a blood glucose level of >250 mg/dL.

### Pressure Ulcer Induction

Under gaseous anesthesia, the animals were shaved on the back and the area was thoroughly cleansed to prevent skin irritation. A fold of skin was raised and placed between two magnetic disks (anisotropic ferrite, 12 mm diameter and 5.0 mm thickness, with an average weight of 2.4 g and 1000 G magnetic force; Algamagnetic, Italy) to create a bridge of skin approximately 1 cm thick.^49^ Based on the results of our previous experiments,^21,22^ we opted for three ischemia–reperfusion (I/R) cycles, with a single I/R cycle involving the application of the magnets for 12 h, followed by a rest period of 12 h without magnets. Following the third application of the magnets, the wounds were covered with a Tegaderm dressing (Tegaderm Roll, 3M). Three days after the final I/R cycle, wound curettage was performed to remove dead/ischemic tissue.

### Group Composition

The following groups were included in this study: db/db mice in whom a pressure ulcer (PrU) was induced, treated with vehicle (*N* = 6); db/db mice in whom PrU was induced, treated with GM18 solution (0.243 mg/ulcer in 50 μL, corresponding to a dosage of 0.243 mg of GM18) (*N* = 6); db/db mice in whom PrU was induced, treated with PLLA15GM18 (1 cm^2^ for each ulcer, corresponding to a dosage of 0.243 mg of GM18) (*N* = 6).

GM18 solution was administered through the Tegaderm dressing into the wound bed using a 1 mL syringe with a 25-gauge needle and repeated every second day. PLLA-GM18 was applied directly to the wound bed following wound curettage (on day 3 after the last I/R cycle), covered with Tegaderm, and the dressing was changed every second day. Saline solution (NaCl 0.9%) was used as vehicle. Each treatment was performed under gaseous anesthesia. No infections were observed during the study.

### Sacrifice and Tissue Sampling

Fourteen days after wound medication began, corresponding to around 50% of wound closure, the mice were deeply anesthetized (isoflurane 3% plus 2 L/min O_2_) and skin samples (1 cm × 1 cm) were taken from the area of the wound. The right-hand wound from each treated animal was collected for morphological analysis, while the left-hand wound was collected for RNA assay.

The left-hand wound from each animal was collected using an 8 mm punch, immediately frozen in liquid nitrogen, and stored at −80 °C until use.

### Histology

A 1 cm^2^ skin sample containing the wound was fixed in paraformaldehyde 4% (v/v) and picric acid-saturated aqueous solution in Sörensen buffer 0.1 M pH 7 for 48 h, embedded in paraffin, and 4 mm thick sections were stained (hematoxylin and eosin, HE).

### Tissue Reverse Transcription Polymerase Chain Reaction (RT-PCR) for ECM Encoding Genes

A pathway-focused gene expression analysis using the RT2 Profiler PCR Arrays (Qiagen), including 84 ECM encoding genes, was performed in skin sampled at 50% of the repair process. RNA was extracted from all animals (six animals per group), quantified (NanoDrop 2000 spectrophotometer), and pooled (100 ng per animal); 600 ng of RNA per group was therefore used for the reverse transcription. Pooled RNAs were retrotranscribed using the RT2 First Strand Synthesis Kit (Qiagen) according to the manufacturer’s instructions, and each pooled group was tested using a single PCR array, using the CFX96 real-time PCR instrument (BioRad). Qiagen mouse extracellular matrix and adhesion protein (PAMM-013Z) relative gene expression was calculated using the 2^–(ΔΔCq)^ comparative method, and a twofold regulation was used as threshold, with the same threshold used for all of the plates. The dedicated Qiagen online data analysis software for the relative quantification of gene expression was used to perform the analysis and generate the graphs.

### Cell Culture

The human BJ fibroblast cell line (ATCC CRL-2522, Manassas, VA) was cultured under standard conditions, in minimum essential medium (MEM, Gibco, Waltham, MA) containing 5.5 mM d-glucose, supplemented with 10% heat-inactivated fetal bovine serum (FBS, Thermo Fisher Scientific, Waltham, MA), 1% penicillin/streptomycin ((100 U/mL)/(100 μg/mL); Thermo Fisher Scientific), in a humidified incubator of 5% CO_2_, at 37 °C. When 70–80% confluency was reached, the cells were split by trypsinization and subcultured in 25 or 75 cm^2^ flasks, at a density of 10 × 10^3^ cells/cm^2^.

Pooled primary human umbilical vein endothelial cells (HUVEC; GIBCO, Invitrogen cell culture. Cat. no. C-015-5C, Waltham, MA) were cultured in phenol red-free basal medium M200 (Life Technologies), containing 10% FBS (Life Technologies, Waltham, MA), 1% Pen/Strep, 1% l-glutamine and growth factors (LSGS, Life Technologies) supplemented with low serum growth supplement (LSGS) containing fetal bovine serum (2%), hydrocortisone (1 μg/mL), human epidermal growth factor (10 ng/mL), basic fibroblast growth factor (3 ng/mL), and heparin (10 μg/mL), in a humidified incubator of 5% CO_2_, at 37 °C. When 70–80% confluency was reached, the cells were passaged by trypsinization and subcultured in 25 or 75 cm^2^ flasks at a density of 7.5 × 10^3^ cells/cm^2^.

Normal adult human primary epidermal keratinocytes were purchased from the American Type Culture Collection (ATCC PCS-200-011). Cells were grown in dermal cell basal media (ATCC PCS-200-030) supplemented with Keratinocyte growth kit components (ATCC PCS-200-040) which contained the following growth components: 0.4% bovine pituitary extract (BPE), 0.1% rh TGF-α (0.5 ng/mL), 3% l-glutamine (6 mM), 0.1% hydrocortisone hemisuccinate (100 ng/mL), 0.1% rh insulin (5 mg/mL), 0.1% epinephrine (1.0 mM), 0.1% apo-transferrin (5 mg/mL) (ATCC PCS-200-400), and 0.1% penicillin–streptomycin ((100 U/mL)/(100 μg/mL)) (Thermo Fisher Scientific), in a humidified cell culture incubator with 5% CO_2_ at 37 °C. When 70–80% confluency was reached, the cells were passaged by trypsinization and trypsin neutralizing solution and subcultured in 75 cm^2^ flasks, at a density of 5 × 10^3^ cells/cm^2^.

### Cell Adhesion Assay on GM18 Coating

HUVEC, FBJ, and HEK cells were seeded on a GM18 coating at three different concentrations (5, 10, and 20 ng/mL). The coating was produced by the deposition of the molecule on the surface of the plate for 24 h in PBS. After seeding in the cell culture incubator at 37 °C and 5% of CO_2_, the cultures were fixed at specific time points (2, 4, and 6 h), using cold 4% paraformaldehyde for 20 min. The nuclei were then stained by incubating the fixed cells with the nuclear staining Hoechst 33258 (1 μg/mL; Thermo Fisher Scientific) for 20 min at room temperature, and washed twice with PBS.

The stained cells were analyzed with the cell-based high-content screening technology using the Cell Insight CX5 instrument (HCS, Thermo Fisher Scientific), using the compartmental analysis tool of the dedicated software. Setting the fluorescence thresholds of the nuclear staining and a proper segmentation algorithm allows the software to recognize every single nucleus and thus count the total number of cells in each well.

### Cell Adhesion Assay Following GM18 Pretreatment

FBJ cells were seeded in 96-well plates at a density of 10 000 cells/well and treated for 24 h with vehicle (dimethyl sulfoxide (DMSO) 1%) or three different doses of GM18 (5, 10, and 20 μg/mL). After 20 min in the cell culture incubator at 37 °C and 5% of CO_2_, the cells were fixed using cold 4% paraformaldehyde for 20 min. The nuclei were then stained by incubating the fixed cells with the nuclear staining Hoechst 33258 (1 μg/mL; Thermo Fisher Scientific) for 20 min at room temperature and washed twice with PBS.

The stained cells were analyzed with the cell-based high-content screening technology using the Cell Insight CX5 instrument (HCS, Thermo Fisher Scientific), using the compartmental analysis tool of the dedicated software. Setting the fluorescence thresholds of the nuclear staining and a proper segmentation algorithm allows the software to recognize every single nucleus and thus count the total number of cells in each well.

### Cell Morphology on PLLA-GM18 Scaffolds and Scanning Electron Microscope Imaging

FBJ cells were seeded on PLLA or PLLA15GM18 and, after 3 days, cultures were fixed and processed for the scanning electron microscopy acquisition.

Scaffolds were mounted in CellCrown24 inserts (Scaffdex, Näsilinnankatu, Finland) and placed in the 24-well plates and cells were seeded directly on scaffolds with a density of 30 × 10^3^ cells/well.

Cultures were fixed using 2.5% glutaraldehyde in cacodylate tampon 0.1 M, pH 7.4, for 1 h at room temperature. Samples were then dehydrated in a graded ethanol series ranging from 50 to 100% with a 5 min incubation time for each step. After immersing the dehydrated scaffolds in hexamethyldisilazane for 10 min, the samples were air-dried.

The dried samples were sputter-coated with gold; then, SEM imaging was performed at 500× and 1500× magnification using an INCAx-sight 7060 apparatus, with an accelerating voltage of 15 kV.

### RNA Extraction, cDNA Synthesis, and Gene Expression Analysis of Cell Cultures

The RNeasy Micro Kit (Qiagen, Milan, Italy) was used to extract the total RNA from the different cell lines cultured in 24-well plates and treated for 24 h with vehicle (1% DMSO) or the three different GM18 concentrations (5, 10, 20 μg/mL). RNA was eluted in RNase-free water, and the concentration was measured through absorbance reading using the NanoDrop 2000 spectrophotometer (Thermo Fisher Scientific). First-strand cDNAs were obtained using the iScript cDNA Synthesis Kit (BioRad) (42 °C for 30 min). Genomic DNA elimination was performed both during the RNA extraction, using on column DNase digestion, and during the cDNA synthesis. An RNA sample with no-reverse transcriptase enzyme in the reaction mix was processed as a no-reverse transcription control sample.

Semiquantitative real-time PCR was performed using the CFX96 real-time PCR system (BioRad, CA). The reactions were performed in a final volume of 20 μL consisting of SYBR Green qPCR master mix (BioRad), 0.4 μM forward and reverse primers, and nuclease-free water. The nonreverse transcriptase enzyme control sample was processed in parallel with the others and tested by real-time PCR for every pair. All primers were designed using Primer Blast software (NCBI, MD) and synthesized by IDT (Coralville, IA): *GAPDH* (Fw: 5′-TCATCCCTGCCTCTACTG-3′; Rev: 5′-TGCTTCACCACCTTCTTG-3′); *ITGA4* (Fw: 5′-GGAAAGAATCCCGGCCAGAC-3′; Rev: 5′-TATGCCCACAAGTCACGATGG-3′); *ITGB1* (Fw: 5′-GGACACAGCCAACAACCCAC-3′; Rev: 5′-AGGAGGCATTCTGGGACAAAG–3′).

### Statistical Analysis

The results of the in vitro experiments were derived from triplicate cell experiments, expressed as mean ± standard error (SEM), and plotted on graphs. Statistical analyses were performed with Prism software (GraphPad), using the Student’s *t*-test to compare the two groups: one-way ANOVA followed by Tukey’s multiple comparisons test for dose–response experiments and two-way ANOVA followed by Dunnett’s multiple comparisons test for time- and treatment-response experiments (see results and key to figures for details).

Results were considered significant when the probability of their occurrence as a result of chance alone was less than 5% (*p* < 0.05).

## Conclusions

This study provides in vivo evidence that the α_4_β_1_ ligand GM18 ameliorates the wound healing process in a mouse chronic wound model by acting on fibroblasts. In vitro data included in the study support a dual action at the α_4_β_1_ binding site: one directed at the cell–matrix interaction, the other at the outside-down mechanism. Despite the single time point of our in vivo study, its main limitation, this study demonstrates that the in vivo modulation of integrin–matrix binding exerted by a combined therapeutic product—one which includes molecules and biomaterial as drug delivery solution—may constitute part of the therapeutic discovery pipeline for chronic skin wounds.

## Supporting Information Available

Detailed description of the GM18 synthesis; differential scanning calorimetry analysis of the PLLA and PLLA-GM18 electrospun scaffolds (Figures S1 and S2, Table S1); release studies (Figure S3); full list of the genes included in the RT2 PCR array PAMM-013ZA (Table S2); clustergram of the whole analyzed gene set (Figure S4); scatter plot analysis of the relative gene expression regulation of tissues treated with GM18-soluble molecule or PLLA15GM18 (Figure S5); comparison between the upregulated and downregulated genes in soluble GM18- and PLLA15GM18-treated compared to vehicle-treated tissues (Figure S6) (PDF)
