# Supplementary material for: Poly(l-lactic acid) Scaffold Releasing an α4β1 Integrin Agonist Promotes Nonfibrotic Skin Wound Healing in Diabetic Mice
Source: ACS Appl Bio Mater. 2022 Dec 21;6(1):296–308. doi: 10.1021/acsabm.2c00890 (PMC9937562; doi:10.1021/acsabm.2c00890)
Supplement: Supplementary file 1 — mt2c00890_si_001.pdf [file mt2c00890_si_001.pdf]

Supporting Information

Poly(L-lactic acid) scaffold releasing an $\alpha_4\beta_1$ integrin agonist promotes non-fibrotic skin wound healing in diabetic mice

Vito Antonio Baldassarro,†[a,b] Valentina Giraldi,†[b] Alessandro Giuliani,[a] Marzia Moretti,[a] Giorgia Pagnotta,[c] Alessandra Flagelli,[b] Paolo Clavenzani,[a] Luca Lorenzini,[a,b] Luciana Giardino,[a,b,d] Maria Letizia Focarete,[b,c] Daria Giacomini,*[b,c] and Laura Calzà* [b,d,e]

[a.] *Department of Veterinary Medical Science, University of Bologna, 50 Via Tolara di Sopra, 40064, Ozzano Emilia, Bologna, Italy.*
[b.] *Interdepartmental Center for Industrial Research in Health Sciences and Technologies, University of Bologna, 41/E Via Tolara di Sopra, 40064, Ozzano Emilia, Bologna, Italy.*
[c.] *Department of Chemistry "Giacomo Ciamician" and INSTM UdR of Bologna, University of Bologna, 2 via Selmi, 40126, Bologna, Italy.*
[d.] *IRET Foundation, 41/E Via Tolara di Sopra, 40064, Ozzano Emilia, Bologna, Italy.*
[e.] *Department of Pharmacy and BioTechnology, University of Bologna, 15 Via San Donato, 40127, Bologna, Italy*
† These authors contributed equally.

* Corresponding authors: D.G. for chemistry experiments, email: daria.giacomini@unibo.it; L. C. for in vitro and in vivo testing, email: laura.calza@unibo.it.

Synthesis of GM18

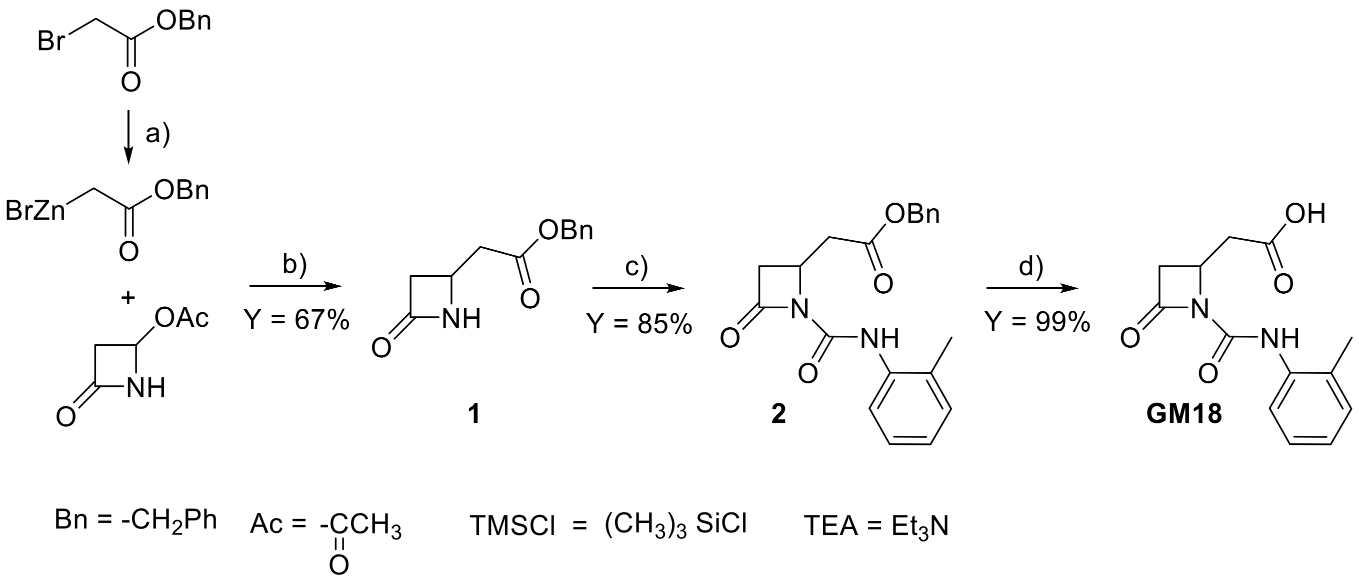

Scheme S1: Reagents and conditions: a) Zn, TMSCl, THF, 32°C, 1 h b) THF, 0°C to rt, 2h c) TEA, o-tolyl isocyanate, CH_2Cl_2, overnight, rt d) H_2, THF/MeOH (1:1), Pd/C 10% mol, 2h, rt.

Detailed procedures

Benzyl 2-(4-oxoazetidin-2-yl)acetate (1) In a 25 mL 3-neck flask under inert atmosphere (N_2), Zn powder (1.412 g, 21.6 mmol, 8 equiv) and anhydrous THF (7 mL) were introduced followed by TMSCl (140 μL, 1.08 mmol, 0.4 equiv). After 30 min of vigorous stirring, the temperature was raised to 30-32 °C and a solution of benzylbromoacetate (1.71 mL, 10.8 mmol, 4 equiv) in THF (13 mL) was slowly added dropwise. After 30 min of stirring at the same temperature the mixture was cooled to rt and decanted. The limpid grey supernatant was then added dropwise at 0°C to a 100 mL flask under nitrogen containing a solution of 4-acetoxy azetidin-2-one (350 mg, 2.7 mmol, 1 equiv) in anhydrous THF (14 mL). The mixture was stirred at rt for 3 h, quenched with a saturated Seignette salt (potassium sodium tartrate) solution and extracted with EtOAc. The organic layers were dried on Na_2SO_4, filtered and concentrated in vacuum. The crude was purified by flash chromatography

(Cyclohexane/AcOEt = 55:45) yielding **1** as a white solid (394 mg, 67%). Spectroscopic data were in fully accordance with those reported in literature. [1]

M.p. 92-95 °C; IR (film, cm^{-1}) 3238, 2961, 1738, 1460, 1372, 1262; ^1H NMR (400 MHz, $CDCl_3$) δ (ppm) 2.64 (dd, J = 9.2, 16.8 Hz, 1H), 2.66 (ddd, J = 1.2, 2.4, 15.2 Hz, 1H), 2.78 (dd, J = 4.8, 16.8 Hz, 1H,), 3.15 (ddd, J = 2.4, 4.8, 15.2 Hz, 1H), 3.96 (dddd, J = 2.4, 4.8, 4.8, 9.2 Hz, 1H), 5.15 (s, 2H), 6.22 (bs, 1H), 7.34-7.39 (m, 5H); ^{13}C NMR (100 MHz, $CDCl_3$) δ (ppm) 39.8, 43.4, 43.8, 66.8, 128.3, 128.5, 128.7, 135.3, 166.9, 170.7; ESI-MS, m/z 220 $[M+H]^+$, 237 $[M+H_2O]^+$, 439 $[2M+H]^+$.

Benzyl 2-(4-oxo-1-(o-tolylcarbamoyl)azetidin-2-yl)acetate (2) In a round bottom flask under inert atmosphere (N_2), TEA (1.05 mL, 7.56 mmol, 5 equiv) was added to a solution of **1** (331 mg, 1.51 mmol, 1 equiv) in anhydrous DCM (13.5 mL). After 15 minutes, o-tolyl isocyanate (0.94 mL, 7.56 mmol, 5 equiv) was added dropwise to the reaction mixture which was subsequently left under stirring at room temperature. At completion (TLC monitoring, 20 h), the reaction was quenched with a saturated aqueous solution of NH_4Cl, and extracted with DCM (3x15 mL). The collected organic layers were dried on Na_2SO_4, filtered and concentrated under reduced pressure. The crude was then re-dissolved in EtOAc at 0°C. After 15 minutes at 0°C, the precipitate was filtered off, the filtrate concentrated under reduced pressure and the crude purified by flash chromatography on silica gel (Cyclohexane/AcOEt = 80:20) yielding **2** as a white solid (451 mg, 85%). Spectroscopic data were in fully accordance with those reported in literature. [1]

M.p. 74−76 °C; IR (film, cm^{-1}) 3338, 1767, 1733, 1713; ^1H NMR (400 MHz, $CDCl_3$) δ (ppm) 2.29 (s, 3H), 2.81 (dd, J = 8.8, 16.5 Hz, 1H), 2.95 (dd, J = 2.8, 16.5 Hz, 1H), 3.36 (dd, J = 6.0, 16.4 Hz, 1H), 3.37 (dd, J = 4.8, 16.4 Hz, 1H), 4.47-4.49 (m, 1H), 5.14 (d, J_{AB} = 12.6 Hz, 1H), 5.18 (d, J_{AB} = 12.6 Hz, 1H), 7.03-7.06 (m, 1H), 7.17-7.23 (m, 2H), 7.32-7.37 (m, 5H), 7.92 (d, J = 8.1 Hz, 1H), 8.42 (bs, 1H); ^{13}C NMR (100 MHz, $CDCl_3$) δ (ppm) 17.7, 37.0, 42.6, 47.5, 66.8, 121.0, 124.4, 126.8, 127.5, 128.3, 128.4, 128.6, 130.4, 135.2, 135.3, 147.8, 166.7, 169.7; ESI-MS m/z 353 $[M + H]^+$, 375 $[M + Na]^+$, 727 $[2M + Na]^+$.

2-(4-oxo-1-(o-tolylcarbamoyl)azetidin-2-yl)acetic acid (GM18) In a 25 mL round bottom flask under inert atmosphere (N_2), Pd/C (18 mg, 10% $^w/_w$) was added to a solution of **2** (180 mg, 0.51 mmol) in a 1:1 mixture of anhydrous THF and methanol (total volume:11 mL). The reaction was then left under stirring in a H_2 atmosphere at room temperature and monitored by TLC. At completion (2 h), the mixture was filtered under vacuum on Celite, washing with methanol. The filtrate was concentrated under reduced pressure and, after titration with pentane, GM18 was obtained as a white solid (131 mg, 99%). Spectroscopic data were in fully accordance with those reported in literature. [1]

M.p. 114−117 °C; IR (film, cm^{-1}) 3344, 1766, 1708; ^1H NMR (400 MHz, $(CD_3)_2CO$) δ (ppm) 2.27 (s, 3H), 2.90 (dd, J = 9.1, 16.7 Hz, 1H), 3.08 (dd, J = 2.9, 16.0 Hz, 1H), 3.26 (dd, J = 3.7, 16.7 Hz, 1H), 3.42 (dd, J = 5.7, 16.0 Hz, 1H), 4.42-4.46 (m, 1H), 7.00-7.04 (m, 1H), 7.17-7.23 (m, 2H), 7.99 (d, J = 8.1 Hz, 1H), 8.55 (bs, 1H); ^{13}C NMR (100 MHz, $(CD_3)_2CO$) δ (ppm) 17.6, 36.6, 43.1, 48.4, 121.2, 124.7, 127.4, 127.7, 131.2, 136.9, 148.8, 168.4, 171.7; ESI-MS m/z 263 $[M + H]^+$, 285 $[M + Na]^+$, 547 $[2M + Na]^+$.

Differential Scanning Calorimetry (DSC) analysis

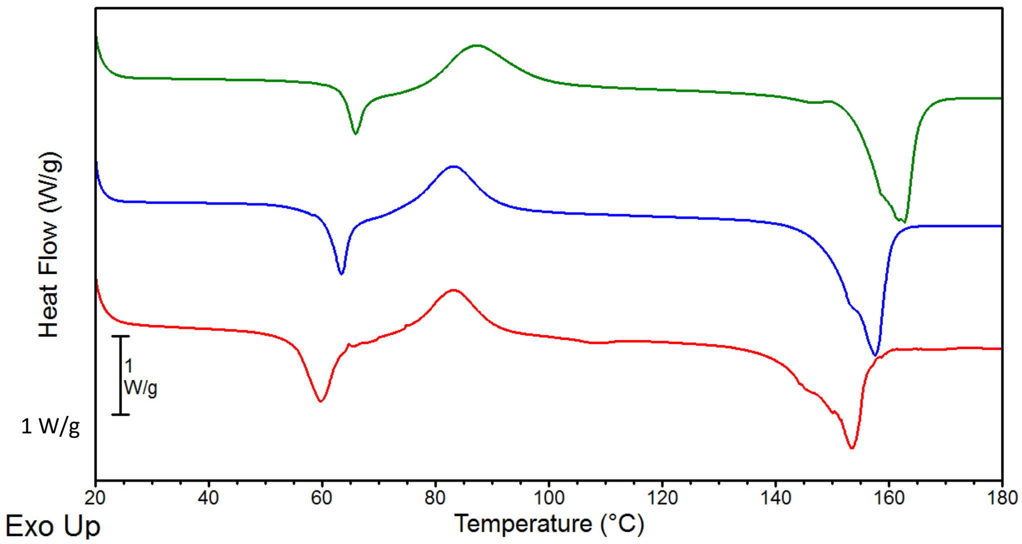

Figure S1: DSC curves of plain PLLA (green), PLLA5GM18 (blue) and PLLA15GM18 (red) (first heating scan, 20°C/min).

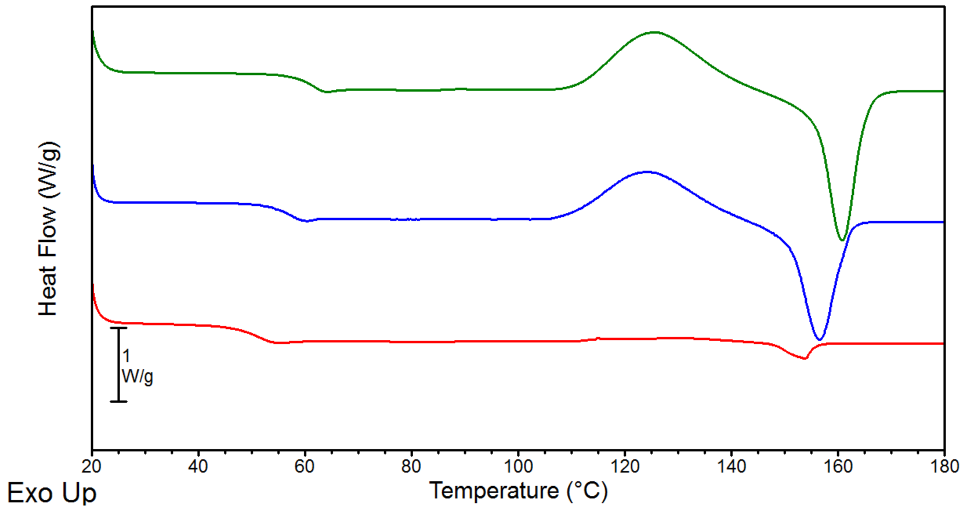

Figure S2: DSC analysis of plain PLLA, PLLA5GM18 and PLLA15GM18 (second heating scan after quench, 20°C/min).

Table S1: Calorimetric properties of PLLA and PLLA-GM18 samples

	Sample	Tg (°C)	ΔCp (J/g °C)	T_c (°C)	ΔH_c (J/g)	T_m (°C)	ΔH_m (J/g)	ΔH_m (PLLA)[a] (J/g)
**First scan**	PLLA	n.d.b	n.d.b	87	37	163	41	41
	PLLA5GM18	n.d. [b]	n.d. [b]	83	36	158	39	39
	PLLA15GM18	n.d. [b]	n.d. [b]	83	33	153	35	35
**Second scan**	PLLA	61	0.60	126	44	161	44	44
	PLLA5GM18	56	0.62	124	37	157	37	42
	PLLA15GM18	50	0.65	130	4	153	4	37

a) Melting enthalpy per gram of PLLA
b) Not detectable due to the presence of aging peak.

Comment to Figure S1, Figure S2 and Table S1

The thermal properties of the electrospun fibers have been investigated by DSC and the results are shown in Figure S1 (first heating scan) and Figure S2 (second heating scan after quench), whereas the calorimetric data are reported in Table S1. In the first heating scan all samples show the effect of the physical aging that appears as an endothermic peak in the glass transition temperature (T_g) region. As expected, this reversible effect is almost partially erased during heating at high temperatures and in the second heating scan the samples show a glass transition whose temperature decreases with the increase of GM18 content. This result can be explained hypothesizing a plasticizing effect of the GM18 molecule. A cold crystallization exothermic peak can be observed in all curves in the temperature range 75-85 °C for the first scan and 100-140°C for the second scan after quench. In the first heating scan, for all the samples, the associated ΔH_c is slightly lower than the ΔH_m (Table S1), indicating that during the electrospinning process a little amount of crystalline phase was developed in the fibers. After quenching from the melt, the cold crystallization exothermic peak was followed by a melting endothermic peak of exactly the same entity for all samples, indicating that the melting phenomena that follows the cold crystallization concerns only the PLLA crystal phase developed during the heating scan, thus demonstrating that completely amorphous PLLA was obtained after quenching, as expected.

Some consideration can be done regarding the influence of the GM18 molecule on the thermal properties of PLLA. The presence of the GM18 component may either simply "dilute" the crystalline phase or to some extent also inhibit PLLA crystallization. In order to answer this question, the melting enthalpy per gram of PLLA present in each sample was calculated (Table S1). Again differences were found for the first and second scan: (i) the values obtained in the first scan were practically constant, showing that, although the overall crystallinity degree of the blends changed in the order PLLA > PLLA5GM18 > PLLA15GM18, the ability for crystallization of PLLA remained unaltered and PLLA crystallized to the same extent as in the pure state, independent of GM18 content; (ii) the values obtained in the second scan, however, showed a great decrease of the crystallinity degree of the samples containing GM18 molecule, becoming very low in the case of PLLA-GM18 15% wt (4 J/g) (Table S1), indicating that the ability for crystallization of PLLA was significantly reduced by the presence of GM18. It can be hypothesized that, after melting of the fibers in the DSC, more stable interactions, and/or more efficient mixing, occurred between PLLA chains and GM18, thus hindering PLLA crystallization.

Release studies

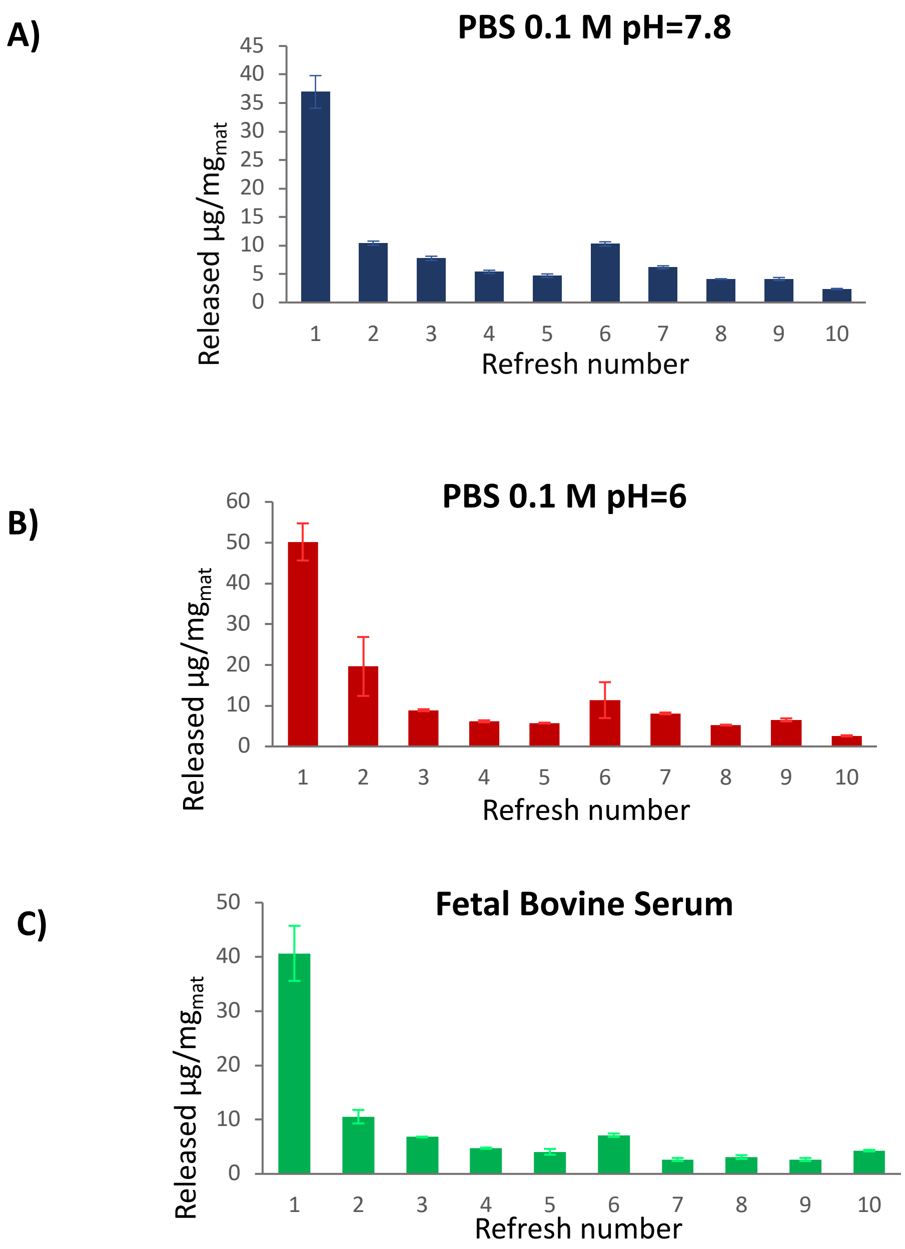

Figure S3: Release of GM18 from PLLA15GM18 scaffold expressed in released µg/mg$_{mat}$ in A) Phosphate buffer solution (PBS) 0.1 M at pH 7.8; B) PBS 0.1 M at pH 6; C) in Fetal Bovine Serum (FBS). Bars represent the mean values ± SD (standard deviation of the means) in triplicate

Table S2. Full list of the genes included in the RT2 PCR array PAMM-013ZA (Qiagen)

Unigene	Refseq	Symbol	Description
Mm.1421	NM_009621	Adamts1	A disintegrin-like and metallopeptidase (reprolysin type) with thrombospondin type 1 motif, 1
Mm.339048	NM_175643	Adamts2	A disintegrin-like and metallopeptidase (reprolysin type) with thrombospondin type 1 motif, 2
Mm.112933	NM_011782	Adamts5	A disintegrin-like and metallopeptidase (reprolysin type) with thrombospondin type 1 motif, 5 (aggrecanase-2)
Mm.100582	NM_013906	Adamts8	A disintegrin-like and metallopeptidase (reprolysin type) with thrombospondin type 1 motif, 8
Mm.423621	NM_009851	Cd44	CD44 antigen
Mm.35605	NM_009864	Cdh1	Cadherin 1
Mm.257437	NM_007664	Cdh2	Cadherin 2
Mm.4658	NM_001037809	Cdh3	Cadherin 3
Mm.184711	NM_009867	Cdh4	Cadherin 4
Mm.470343	NM_007727	Cntn1	Contactin 1
Mm.277735	NM_007742	Col1a1	Collagen, type I, alpha 1
Mm.2423	NM_031163	Col2a1	Collagen, type II, alpha 1
Mm.249555	NM_009930	Col3a1	Collagen, type III, alpha 1
Mm.738	NM_009931	Col4a1	Collagen, type IV, alpha 1
Mm.181021	NM_009932	Col4a2	Collagen, type IV, alpha 2
Mm.389135	NM_007734	Col4a3	Collagen, type IV, alpha 3
Mm.7281	NM_015734	Col5a1	Collagen, type V, alpha 1
Mm.2509	NM_009933	Col6a1	Collagen, type VI, alpha 1
Mm.390287	NM_010217	Ctgf	Connective tissue growth factor
Mm.384762	NM_009818	Ctnna1	Catenin (cadherin associated protein), alpha 1
Mm.34637	NM_009819	Ctnna2	Catenin (cadherin associated protein), alpha 2
Mm.291928	NM_007614	Ctnnb1	Catenin (cadherin associated protein), beta 1
Mm.3433	NM_007899	Ecm1	Extracellular matrix protein 1
Mm.286375	NM_133918	Emilin1	Elastin microfibril interfacer 1
Mm.2824	NM_009848	Entpd1	Ectonucleoside triphosphate diphosphohydrolase 1
Mm.297992	NM_010180	Fbln1	Fibulin 1
Mm.193099	NM_010233	Fn1	Fibronectin 1
Mm.266790	NM_013500	Hapln1	Hyaluronan and proteoglycan link protein 1
Mm.2168	NM_010406	Hc	Hemolytic complement
Mm.435508	NM_010493	Icam1	Intercellular adhesion molecule 1
Mm.5007	NM_008396	Itga2	Integrin alpha 2
Mm.57035	NM_013565	Itga3	Integrin alpha 3
Mm.31903	NM_010576	Itga4	Integrin alpha 4
Mm.16234	NM_010577	Itga5	Integrin alpha 5 (fibronectin receptor alpha)
Mm.96	NM_008399	Itgae	Integrin alpha E, epithelial-associated
Mm.1618	NM_008400	Itgal	Integrin alpha L
Mm.262106	NM_008401	Itgam	Integrin alpha M
Mm.227	NM_008402	Itgav	Integrin alpha V
Mm.22378	NM_021334	Itgax	Integrin alpha X
Mm.263396	NM_010578	Itgb1	Integrin beta 1 (fibronectin receptor beta)

Mm.1137	NM_008404	Itgb2	Integrin beta 2
Mm.87150	NM_016780	Itgb3	Integrin beta 3
Mm.213873	NM_001005608	Itgb4	Integrin beta 4
Mm.303386	NM_008480	Lama1	Laminin, alpha 1
Mm.256087	NM_008481	Lama2	Laminin, alpha 2
Mm.42012	NM_010680	Lama3	Laminin, alpha 3
Mm.27560	NM_008483	Lamb2	Laminin, beta 2
Mm.435441	NM_008484	Lamb3	Laminin, beta 3
Mm.1249	NM_010683	Lamc1	Laminin, gamma 1
Mm.14126	NM_019471	Mmp10	Matrix metallopeptidase 10
Mm.4561	NM_008606	Mmp11	Matrix metallopeptidase 11
Mm.2055	NM_008605	Mmp12	Matrix metallopeptidase 12
Mm.5022	NM_008607	Mmp13	Matrix metallopeptidase 13
Mm.280175	NM_008608	Mmp14	Matrix metallopeptidase 14 (membrane-inserted)
Mm.217116	NM_008609	Mmp15	Matrix metallopeptidase 15
Mm.156952	NM_032006	Mmp1a	Matrix metallopeptidase 1a (interstitial collagenase)
Mm.29564	NM_008610	Mmp2	Matrix metallopeptidase 2
Mm.4993	NM_010809	Mmp3	Matrix metallopeptidase 3
Mm.4825	NM_010810	Mmp7	Matrix metallopeptidase 7
Mm.16415	NM_008611	Mmp8	Matrix metallopeptidase 8
Mm.4406	NM_013599	Mmp9	Matrix metallopeptidase 9
Mm.4974	NM_010875	Ncam1	Neural cell adhesion molecule 1
Mm.433941	NM_010954	Ncam2	Neural cell adhesion molecule 2
Mm.343951	NM_008816	Pecam1	Platelet/endothelial cell adhesion molecule 1
Mm.236067	NM_015784	Postn	Periostin, osteoblast specific factor
Mm.5245	NM_011345	Sele	Selectin, endothelial cell
Mm.1461	NM_011346	Sell	Selectin, lymphocyte
Mm.3337	NM_011347	Selp	Selectin, platelet
Mm.8739	NM_011360	Sgce	Sarcoglycan, epsilon
Mm.291442	NM_009242	Sparc	Secreted acidic cysteine rich glycoprotein
Mm.379020	NM_009262	Spock1	Sparc/osteonectin, cwcv and kazal-like domains proteoglycan 1
Mm.288474	NM_009263	Spp1	Secreted phosphoprotein 1
Mm.289702	NM_009306	Syt1	Synaptotagmin I
Mm.14455	NM_009369	Tgfbi	Transforming growth factor, beta induced
Mm.4159	NM_011580	Thbs1	Thrombospondin 1
Mm.26688	NM_011581	Thbs2	Thrombospondin 2
Mm.2114	NM_013691	Thbs3	Thrombospondin 3
Mm.8245	NM_011593	Timp1	Tissue inhibitor of metalloproteinase 1
Mm.206505	NM_011594	Timp2	Tissue inhibitor of metalloproteinase 2
Mm.4871	NM_011595	Timp3	Tissue inhibitor of metalloproteinase 3
Mm.454219	NM_011607	Tnc	Tenascin C
Mm.76649	NM_011693	Vcam1	Vascular cell adhesion molecule 1
Mm.158700	NM_001081249	Vcan	Versican
Mm.3667	NM_011707	Vtn	Vitronectin
Mm.328431	NM_007393	Actb	Actin, beta
Mm.163	NM_009735	B2m	Beta-2 microglobulin

Mm.309092	NM_008084	Gapdh	Glyceraldehyde-3-phosphate dehydrogenase
Mm.3317	NM_010368	Gusb	Glucuronidase, beta
Mm.2180	NM_008302	Hsp90ab1	Heat shock protein 90 alpha (cytosolic), class B member 1

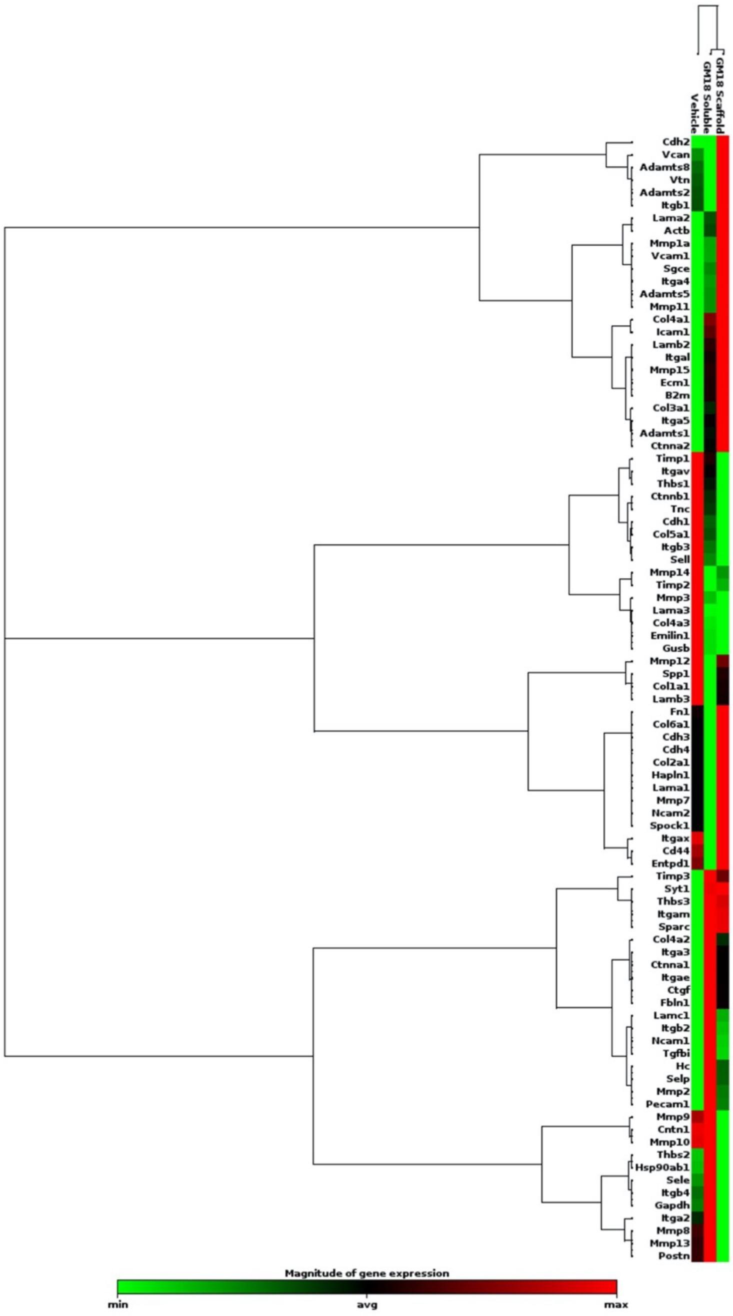

Figure S4. Clustergram of the whole analyzed gene set. The magnitude of expression is indicated with a color code between the maximum (red) and the minimum (green) within the same gene across the groups.

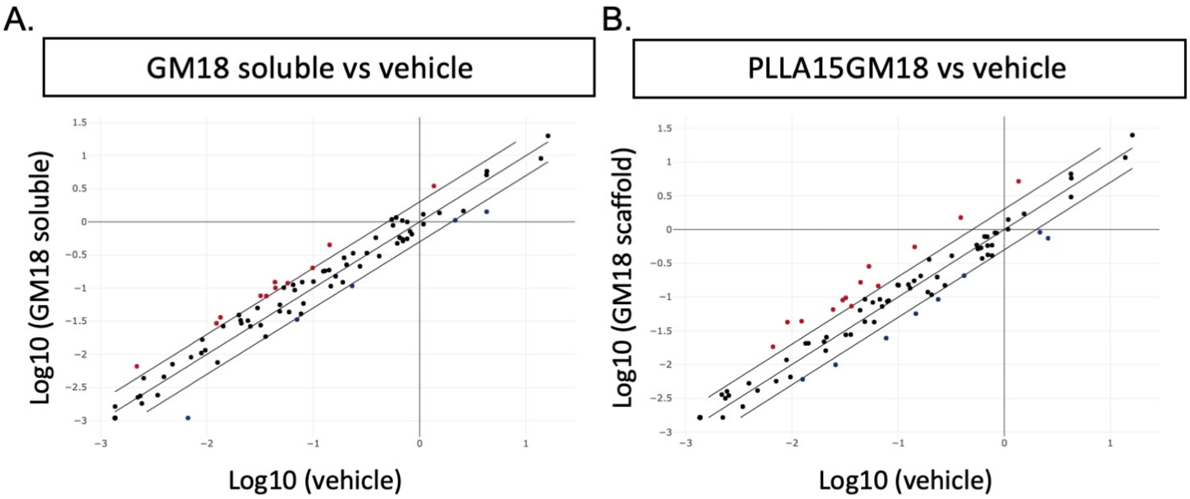

Upregulated		Dwonregulated	
**Gene Symbol**	**Fold Regulation**	**Gene Symbol**	**Fold Regulation**
Adamts1	**2.28**	*Itgax*	**-2.10**
Col4a1	**3.15**	*Mmp12*	**-2.15**
Col4a2	**2.06**	*Mmp3*	**-2.04**
Ctnna1	**2.04**	*Spp1*	**-3.00**
Hc	**3.02**	*Vtn*	**-6.04**
Icam1	**2.39**		
Itgal	**2.39**		
Pecam1	**2.82**		
Selp	**2.69**		
Thbs3	**2.09**		
B2m	**2.56**		

Upregulated		Downregulated	
**Gene Symbol**	**Fold Regulation**	**Gene Symbol**	**Fold Regulation**
Adamts1	**3.73**	*Cdh1*	**-3.13**
Adamts5	**5.37**	*Mmp10*	**-2.60**
Col4a1	**3.87**	*Mmp13*	**-2.57**
Icam1	**3.03**	*Mmp3*	**-2.38**
Itga4	**2.67**	*Sell*	**-2.09**
Itgal	**3.55**	*Thbs1*	**-2.02**
Lama2	**3.00**	*Timp1*	**-2.59**
Lamb2	**2.25**	*Tnc*	**-3.48**
Mmp1a	**3.87**		
Sgce	**4.67**		
Thbs3	**2.01**		
Vtn	**2.76**		
B2m	**3.80**		

Figure S5. Graph showing the scatter plot of the relative gene expression regulation of tissues treated with GM18 as a soluble molecule (A) or PLLA15GM18 (B) versus tissues treated with vehicle, with a cut-off value of fold of changes > 2. The regulated genes, with the relative fold of change values, are shown in the relative tables, indicating up-regulated (red) and down-regulated (blue) genes in GM18 treated tissues compared to vehicle.

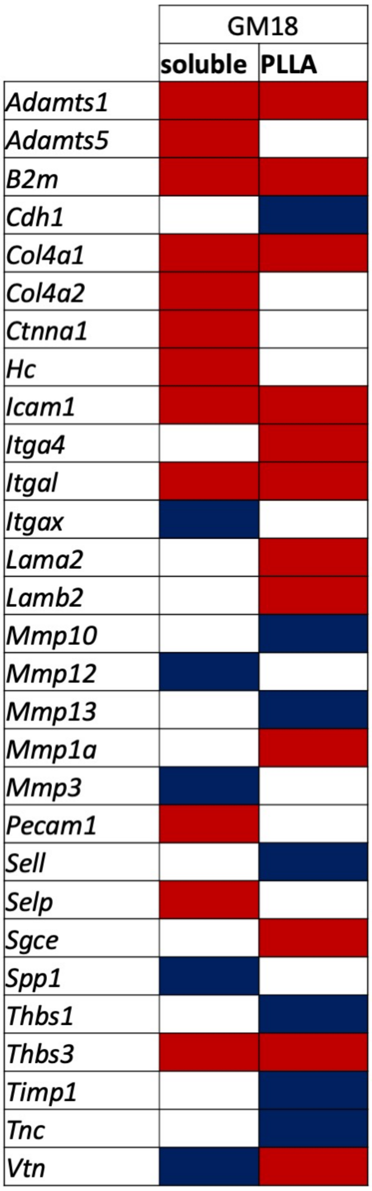

Figure S6. Comparison between the up-regulated (red) and down-regulated (blue) genes in soluble GM18 and PLLA15GM18 treated- compared to vehicle-treated tissues.

References

[1] Baiula M., Galletti P., Martelli G., Soldati R., Belvisi L., Civera M., Dattoli S.D., Spampinato S.M. and Giacomini D. “New β-lactam derivatives modulate cell adhesion and signaling mediated by RGD-Binding and leukocyte integrins” *J. Med. Chem*, **2016**, 59, 9721-9742; doi: 10.1021/acs.jmedchem.6b00576.

[2] Martelli G., Bloise N, Merlettini A, Bruni G, Visai L, Focarete ML, Giacomini D. Combining Biologically Active β-Lactams Integrin Agonists with Poly(l-lactic acid) Nanofibers: Enhancement of Human Mesenchymal Stem Cell Adhesion. Biomacromolecules. 2020 Mar 9;21(3):1157-1170. doi: 10.1021/acs.biomac.9b01550. Epub 2020 Feb 14. PMID: 32011862; PMCID: PMC7997109.
